# Influence of Roughness on Shear Bonding Performance of CFRP-Concrete Interface

**DOI:** 10.3390/ma11101875

**Published:** 2018-10-01

**Authors:** Yushi Yin, Yingfang Fan

**Affiliations:** 1 Department of Civil Engineering, Dalian Maritime University, Dalian 116026, China; fanyf@dlmu.edu.cn; 2 Liaoning Provincial College of Communications, Shenyang 110122, China

**Keywords:** bonding stress, concrete, CFRP, effective bond length, interface, single shear test

## Abstract

The potential of Fiber Reinforced Polymer (FRP) in the reinforcement of concrete structures has been shown in many studies and practical applications. However, few works have focused systematically on the development of quantitative criteria to measure surface roughness and relate this parameter to the bonding mechanical property. Moreover, some researchers have declared that, if the concrete interface is rougher, the bond performance between FRP and concrete will be increase, However, there is no answer to how rough the surface is. There are limited application standards for engineers to conduct in FRP reinforcement projects. This work evaluated several concrete specimens with three different strengths and six types of interface roughness. A single shear test was conducted to study the influence of surface roughness on the interfacial bonding performance of a carbon fiber-reinforced composite (CFRP)-concrete beam. The results show that, among the six interfaces, a concrete interface with the roughness of 0.44 has the best interfacial bonding performance. An interfacial appearance with the cement mortar almost cleaned away, and almost one fifth of the single coarse aggregate bared will get the best bond performance. Roughness parameters significantly influenced the effective bond length. The effective bond length of the six interfaces experienced an overall decreasing trend as the roughness increased. The bond–slip curves of concrete interfaces with roughness of 0.25–0.44 did not significantly change the rigidity within the brittle region. The rougher the interface was, the shorter the brittle region was. After entering a plasticity stage, the bond–slip curves for the six types of interfaces all declined with different slopes, and the max slip values were 0.04–0.35 mm when debonding failure occurred.

## 1. Introduction and Background

The use of Fiber Reinforced Polymer (FRP) materials to strengthen reinforced concrete elements has gained more and more popularity in the last decades, for their relevant properties such as the higher strength to weight ratio and the excellent corrosion resistance. In this strengthening method, the performance of the FRP-to-concrete interface in providing an effective stress transfer is of crucial importance. Indeed, several failure modes in FRP-strengthened RC members are directly caused by interfacial debonding between the FRP and the concrete. Standards from many countries are proposed about how to paste FRP to concrete surface; in more details, surface preparation is the process by which the concrete substrate must be sound, clean, and suitably roughened. This process includes removing the unsound concrete and bond-inhibiting films, strength verification and opening of the pore structure.

Some of the most common surface preparation methods are: steel brushing, angle grinding, sand blasting, water–sand mixed spray, high-pressure water spray, and chiseling [1,2]. The rough surfaces formed through these methods are not obvious. The chisel method can produce an obvious uneven surface with significant roughness. Chajes [3] noted that mechanical polishing improved interfacial bonding strength. Yao et al. [4] used single shear tests to demonstrate that interface processing changed bonding strength significantly. Delaney and Karbhari [5] found that interface processing influenced both the structure’s strength and stability. In China, Li et al. [6] polished and generated interfaces with three types of roughness using sand papering and mechanical polishing. The polished interfaces were compared with the unpolished interfaces to analyze the influence of interface roughness on the ultimate load and displacement. Unfortunately, to date, extensive experimental data concerning the FRP to concrete bond quality are available mainly for sand blasting while, for others treatments, few data can be found.

As a design level, construction guidance relevant to strengthening projects in different countries have confirmed that interface pre-processing positively influences strengthening behavior [7,8]. In 1997, the International Concrete Repair Institute (ICRI) published a report addressing whether interface processing benefits the performance of strengthened beams. In 2002, the American Concrete Institute (ACI) recommended using CSP3 concrete surfaces polished with an angle grinder and sandblast method as construction standards. However, practice demonstrates that, apart from the ICRI standard and methods proposed by ACI, if the concrete’s surface roughness is formed, the interfacial bonding performance can always be strengthened. For example, one handbook on concrete compiled by the Bureau of Reclamation, U.S. Department of Interior requires that, before recasting concrete, the bad, loose, and cast-in-place concrete should be thoroughly removed using an iron chisel or other applicable tools. Sand blasting with a water spray gun, air rock drill, or other applicable methods are used to roughen, clean up, and dry the concrete [9]. In Specifications for Strengthening Design of Highway Bridges (JTG/TJ22-2008) in China [10], the interfacial roughness of reinforcing structures is required to be 6 mm or more. However, the key operation technique for forming the 6 mm roughness is not provided. These facts show that interfaces with appropriate roughness are good for the strengthening of the bonding stress of CFRP-concrete.

Once the importance of the surface roughness is recognized, the following fundamental issue is the achievement of a uniform, as well as easy and convenient, roughness parameter for engineers to conduct in the project on sites.

In this perspective, the present paper describes the results of an experimental program aimed at investigating, from a qualitative view point, the roughness effects on the interface bond performance. Several CFRP-concrete specimens were prepared with six different surface roughness levels, strengthened by CFRP sheets and then tested. Prior to CFRP being pasted, surface roughness was measured by means of Chinese sand-filling method, which proved to be easy and valid. A single shear test was carried out on the CFRP-concrete specimens, and several spaced strain gauges were placed along the CFRP sheet to measure strains.

Results show a significant effect about concrete surface roughness on the interfacial bond performance between CFRP and concrete. The failure mode, the effective bond length and the interface law are also affected by the type of the surface roughness.

## 2. Experimental Setup

### 2.1. Specimens Preparation

The concrete used in the tests consisted of commercial portland ordinary cement 42.5(P.O 42.5 cement), produced by a plant operated by the Jilin Yatai Group in China. The fly ash was Level I superfine fly ash produced by Lianyungang Power Plant in China. The aggregate was medium sand with a fineness modulus of 2.5 and continuous graded artificial gravel with particle sizes ranging 5–20 mm. The additive was a superplasticizer produced by Hongxiang Building Additive Plant from Shenyang in China, with a water-reducing rate (mass fraction) of 18%. The water was running water appropriate for domestic use. The CFRP was a HICOMA-HITEX series carbon fiber sheet produced by Nanjing Hitech Composites Co., Ltd from Nanjing in China. The adhesive colloid was an epoxy resin AB adhesive, with the bonding resin compounded at a mass ratio of 2:1. Table 1 and Table 2 show the raw material properties.

**Figure 1:**
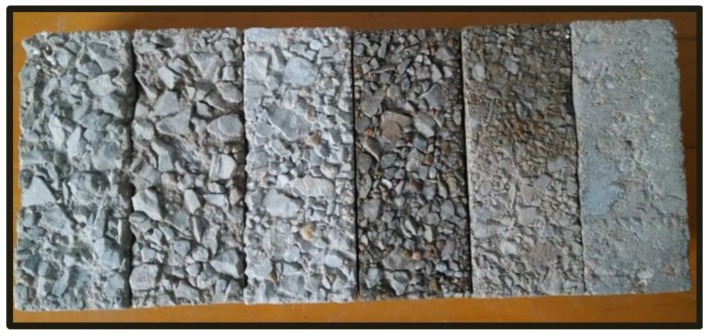
Concrete surfaces with different roughness.

The concrete specimen was 80 mm × 80 mm × 200 mm in size. To produce the roughness, a layer of retarder was first gently coated on the bottom surface of the concrete specimen mold to reduce the negative influence of cracks formed as a result of concrete damage during the tests. After the test specimen was cast for 24 h and demolded, a steel brush method was used to form six types of interfaces with different roughness and specific discrimination. This helped distinguish the rough surfaces, but also greatly reduced the discretization influence of the concrete’s surface damage on the test results, as shown in Figure 1. The tests involved the use of concrete of three different strengths (C30, C40, and C50). Six levels of interface roughness were formed for each strength grade, and the three test specimens with the same roughness formed a group. There were 54 total test specimens. 

**Figure 2:**
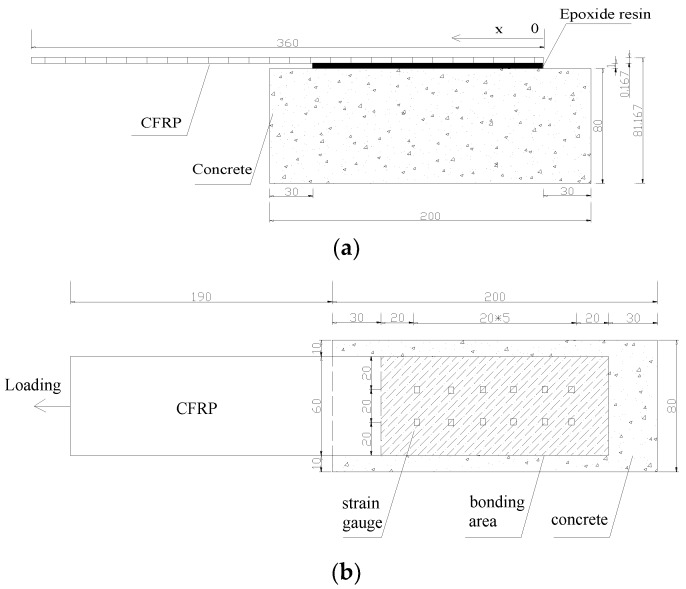
Layout of strain gauges in bonding area: (**a**) side view; (**b**) top view.

Figure 2 shows that a pair of strain gauges were attached within a range of 60 mm × 140 mm on the upper surface of each specimen. The gauges were used to detect the variations in strain along the direction of the bonding length during a single shear test. To weaken the influence produced by the random roughness of interfaces, two rows of strain gauges at 5 mm × 3 mm were arranged at a 20 mm interval along the direction of the interfacial bonding length. The strain value for each cross section was the average of values of the two strain gauges.

### 2.2. Quantification of Roughness

Existing quantitative methods cannot accurately propose specifications from different counties. However, some methods can indirectly determine the interfacial roughness. Specification MC2010 [11] provides some methods that define the interface roughness. This study adopted a sand filling method [12], a common method in China, to measure roughness. The measuring process is: (1) fences are used to block all sides of the concrete bonding area, making the highest points of fences level with the highest point of the concrete uneven surface; (2) standard sand is then poured into the space enclosed by the fences and a spatula is used to smooth the sand that is higher than the fences; and (3) the fences are removed, all the enclosed sand is poured out and its volume is measured. The average height h of pumped sand can be expressed by volume V of standard sand divided by the concrete’s bonding area, as shown in Formula (1):

(1) $$h=\frac{V}{\mathrm{ab}}\;$$

where a and b are the length and width of the concrete’s bonding surface, respectively.

Roughness fi is calculated using Formula (2) [13]:

(2) $$\mathrm{fi}=\frac{h}{\delta }\;$$

where *δ* represents the maximum value of the bump depth of the concrete’s bonding surface. 

### 2.3. Method to Measure δ

**Figure 3:**
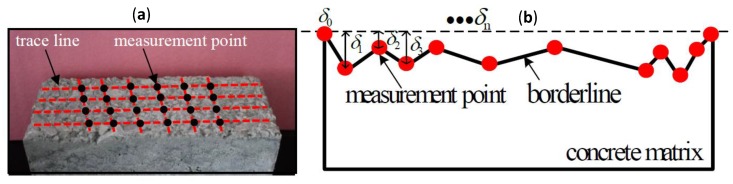
Trace lines, measurement points and getting points in the bond area. (**a**) the total measurement points for one specimen; (**b**) a contour line in the rough concrete surface.

The bonding area for the test was $60\times 140\;{\mathrm{mm}}^{2}$. To carefully illustrate the bump depth value for each position, a 60 mm length with 10 mm increments from both sides was divided into three equal sections along the direction of concrete bonding length. Four trace lines were formed, as shown in Figure 3.

**Figure 4:**
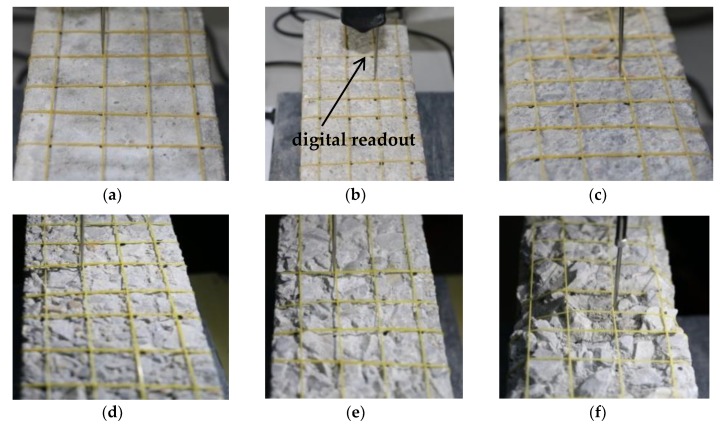
Bump depth values for acquisition points for the digital readout micrometer. (**a**) f0; (**b**) f1; (**c**) f2; (**d**) f3; (**e**) f4; (**f**) f5.

There were four trace lines along the bonding length direction, with each trace line having a length of 100 mm. Five equal parts were divided along the length of trace line, and a digital readout micrometer was used to acquire the bump depth value $\delta_{i}(i=1,2\cdots 24)$ at the measurement point positions along the trace line. The maximum value *δ_max_* was used *δ* in Formula (2), as shown in Figure 4. 

Table 3 shows the quantitative values for the roughness of the six types of interfaces.

As Table 3 shows, with the concrete surface becomes rougher, the interfacial quantitative roughness value gets bigger. For interface f0, the maximum value of concrete surface concave to convex is only 0.3 mm, while, for interface f5, the maximum is 20 mm. From f0 to f5, the quantified roughness value increases from 0.25 to 0.88, i.e. the roughness rises by 252%. The reason is that the concave to convex value is the single by single point measurement, and the quantification of roughness is the average depth h of the bond area, thus the sand filling measurement is more accurate and scientific to quantify the interface roughness.

**Figure 5:**
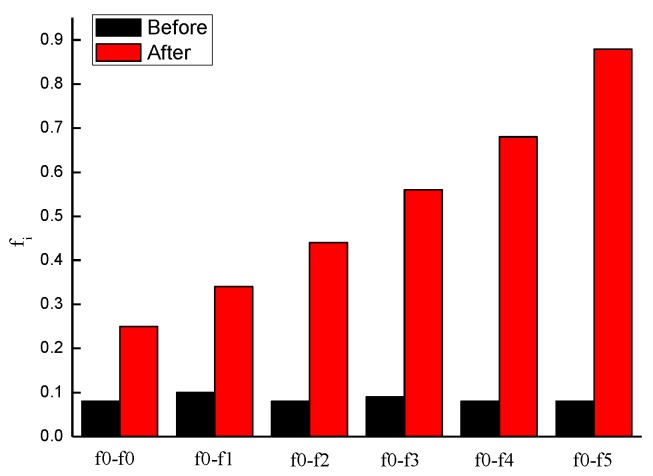
Comparison of roughness before and after interface processing.

In Figure 5, f*i*–f*j* (*i* = 0; *j* = 1, 2, 3, 4, 5) mark the procedure from interface *i* to interface *j*. For example, f0–f2 indicates that, before processing, it was interface f0, and, after processing, it was interface f2. The variable f0–f0 indicates the process of removing the floating mortar with abrasive paper after demolding. Figure 5 shows that, after the artificial brushing, the six types of interfaces become rougher. The roughness levels showed a significant discrepancy: the greater is the value of *j*, the greater is the value of f*i*. Interfaces f0–f5 are roughened by 2.1, 2.4, 4.5, 5.2, 7.5, and 10 times, respectively, with f5 being roughened most significantly.

### 2.4. Test Process

**Figure 6:**
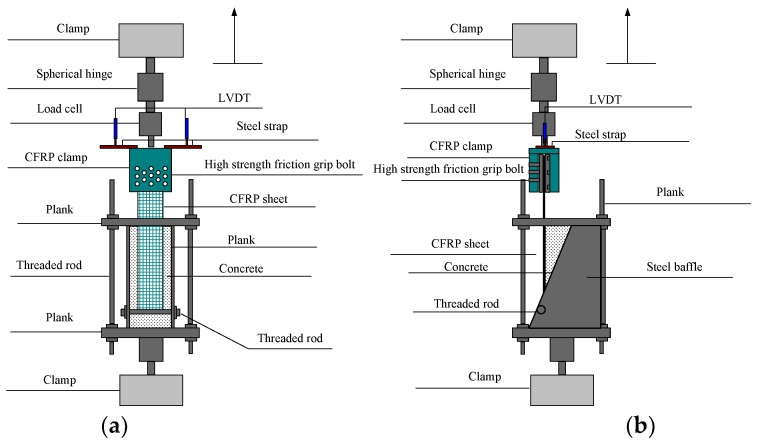
Schematic diagram of the devices for single shear tests: (**a**) front view; and (**b**) side view.

In tests, an electro-hydraulic servo material testing machine was used for loading, with a loading rate of 1 kN/min. Figure 6 shows the device used for single shear tests. In the test process, strain gauges were first connected to the dynamic data acquisition system. To collect the CFRP’s slip, two LVDTs were put on the steel strap. The slip value was taken as the average of the two LVDTs. Every testing machine was connected to the computer, enabling real-time detection of variations in the strain with the loading force. 

## 3. Test Phenomena and Failure Modes

### 3.1. Test Phenomena

In the loading procedure, the load and strain of CFRP sheet near the loading end were synchronously increased. When the load reached 20% of the ultimate bond load, the CFRP sheet began to make slight tearing sounds. When the load continued to increase to 40% of the ultimate bond load, there were discontinuous “snap” sounds as abrupt changes occurred to the CFRP surface strain. This indicated that the CFRP sheet and concrete directly showed the debonding phenomenon. When the load reached 70–80% of ultimate bond load, the debonding sounds were heard more frequently, and this process lasted longer. In the process, some specimens varied in load, however, the variation range was not large. When the load increased to the ultimate bond load, a “snap” was heard and the CFRP debonded from concrete specimens. There was no obvious sign before failure, and the failure was characterized as brittle. 

**Figure 7:**
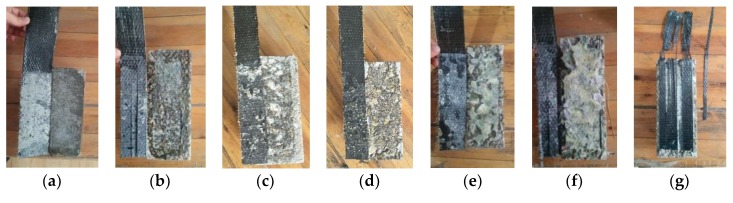
Failure modes of CFRP-concrete interfaces: (**a**) f0; (**b**) f1; (**c**) f2; (**d**) f3; (**e**) f4; (**f**) f5; (**g**) non-ideal failure mode.

**Figure 8:**
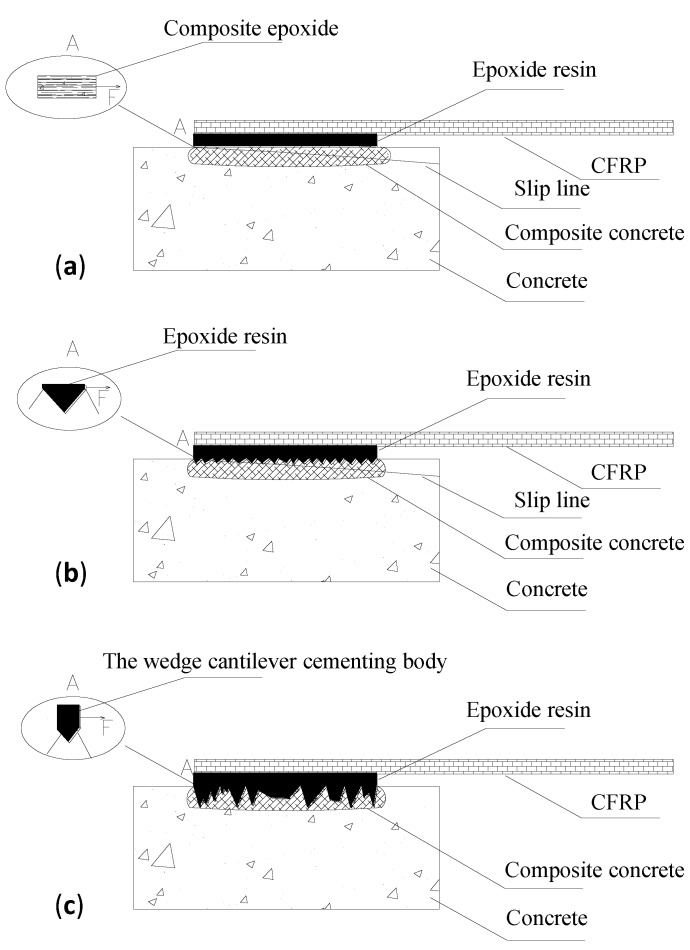
Different interface bond mechanisms. (**a**) Interface a: f0; (**b**) Interface b: f1–f2; (**c**) Interface c: (f3–f5).

After the specimens with six modes of interfaces suffered a single shear failure, they exhibited four failure modes. Figure 7 and Figure 8 show that the first mode of failure occurred when the CFRP sheet was debonded from the interfaces between the adhesive layer and concrete (Figure 7b,d,e,f and Figure 8b,c). The second failure mode was that the CFRP sheet was debonded from the mortar surfaces (Figure 7a and Figure 8a). The third failure mode was that the CFRP sheet was debonded from concrete surfaces, and, at the loading end, triangular wedge concrete was attached (Figure 7c and Figure 8b). The fourth failure mode was a non-ideal failure mode. In this case, the CFRP sheet suffered tearing failure at the loading end because of stress concentration (Figure 7g); this failure mode is not considered in this paper.

### 3.2. Roughness Effects on Interface Bond Mechanism

There are three typical interfaces. Interface a, comprised of the epoxide resin, CFRP sheet and concrete, presents excellent bond performance. Especially when the epoxide resin adhesive penetrates a certain depth through the micro pore of concrete, a new composite is formed combining the epoxide resin and the concrete in this area. Given that the combined epoxide resin and the concrete are jointed together around the coarse aggregate, it is difficult to be broken deep inside the coarse aggregate area, so the slip line is on the surface of the mortar (Figure 8a).

As shown in Figure 8b, with the increasing roughness, the concrete surface presents some coarse aggregates, among which the epoxide resin forms the uniformed epoxide matrix. As the shearing resistance strength of the coarse aggregate is much larger than the strength of the concrete cement layer under Interface a, it takes more time to break than Interface b, while the corresponding failure load is highly required, with a triangular wedge concrete block debonded during the failure.

As for Interface c, the roughness of this interface continues to grow, and the majority of the coarse aggregate is fully revealed. Under such circumstance, the roughness degree of the interface shall be even more serious. The height difference of the bordering concave and convex points are seriously irregular. Hence, the bonding resin fails to bond the CFRP and the concrete at the interface, creating the gaps among them. Additionally, the epoxide resin between the convex aggregates is easy to form the cantilever structure, forming the stress concentration, which will greatly decrease the interface bond property. The bond property of Interface c is extremely low, and, as the concrete strength grows, the failure load of the bond is less influential. However, as the aggregate size increases, for the same rough interface, the bond stress is greatly elevated, which is consistent with the mentioned mechanism.

**Figure 9:**
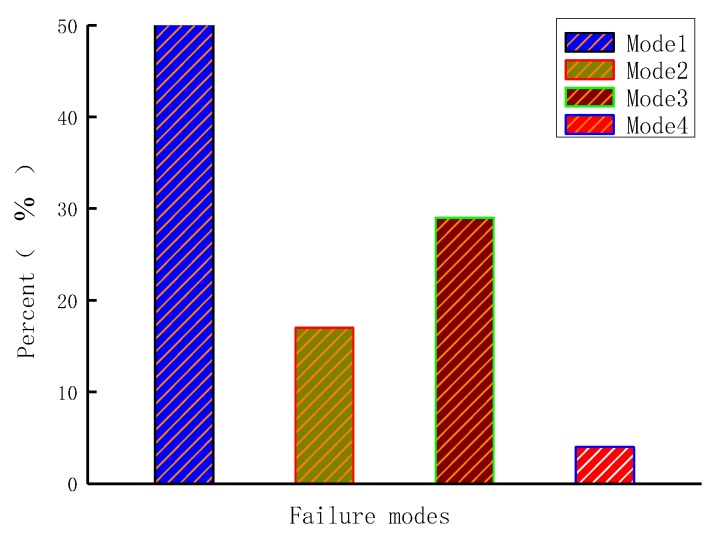
Percentage of different failure modes.

Figure 9 shows that the first type of failure mode was the most common, accounting for 50% of all failure modes. The second type accounted for 17%, the third type accounted for 29%, and the fourth type accounted for 4%.

## 4. The Influence of Roughness on the Bonding Performance of FRP-Concrete Interface

### 4.1. Test Results and Discussion

Single shear test was done for the specimens of f0–f5. Key parameters are shown in Table 4, where each value of the parameter was the average of three specimens.

From the key parameters values $\tau_{m}$, $s_{\tau }$, and $s_{f}$, some conclusions can be made. As the bond strength $\tau_{m}$ gets bigger, the corresponding $s_{\tau }$ and $s_{f}$ both increase, and failure mode changes from 1, 2, and 3 to 1. Mortar debonding from concrete only occurs from the f0 and f1 concrete surfaces. For the roughness levels f3–f5, the failure mode is almost always 1. The key parameters do not influence greatly the failure mode, while the concrete surface roughness plays a significant role in the interface failure mode.

### 4.2. The Influence of Roughness on the Strain–Position Relationship of CFRP Sheet

**Figure 10:**
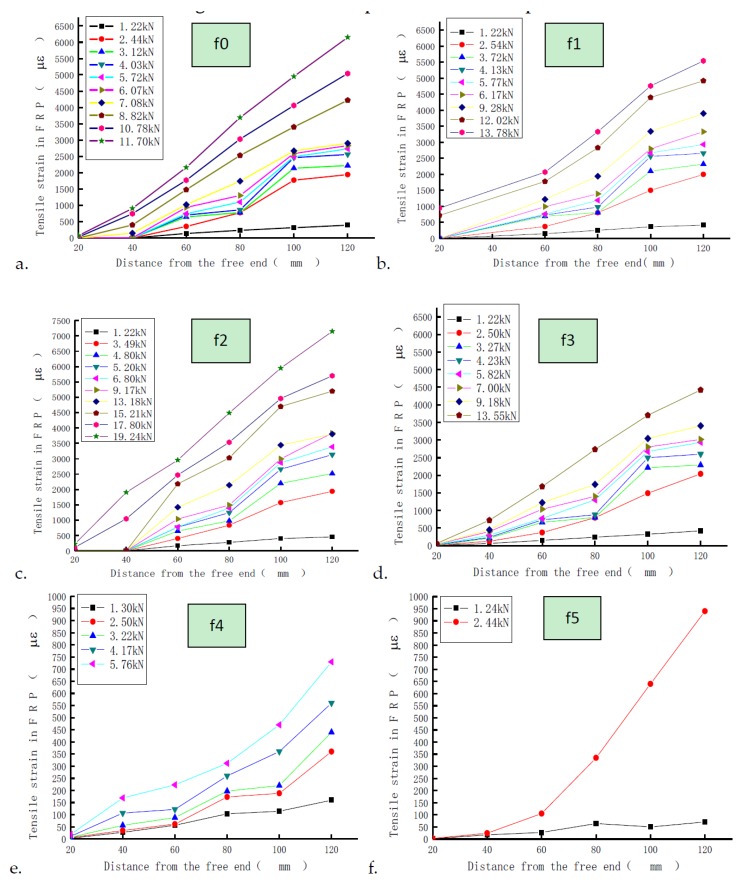
Strain–position relationship of the six types of interface specimens (C40 specimen). (a: f0; b; f1; c: f2; d: f3; e: f4; f: f5).

In Figure 10, the free end is the opposite end from the loading end. Figure 10 shows the strain–position relationship of the CFRP sheet along the direction of the bonding length in the loading procedure for the C40 specimen. On the interfaces of specimens with the roughness of f0–f2, the interfacial bonding shear stress gradually increases as the roughness increases. When the strain gauges at the same position reached the same value, the specimens with greater roughness required greater loading force. For the specimens with a roughness of f3–f5, as the roughness increased, the interfacial bonding shear stress rapidly declined, bringing about a greater loss. 

### 4.3. The Influence of Roughness on Interfacial Effective Bond Length

The bond–slip relationship is the constitutive property determining the bonding performance at the CFRP-concrete interfaces. CFRP strain distribution data were collected based on single shear tests. The bond–slip relationship for the test was obtained using residual calculations.

The local bonding stress *τ_i_* at the position of the *i*th strain gauge is:

(3) $$\tau_{i}=\frac{E_{f}t_{f}(\varepsilon_{i}-\varepsilon_{i-1})}{\Delta x}\;$$

Assuming the interval between strain gauges is ∆*x*, then the slip value *S_i_* at the position of the *i*th strain gauge is:

(4) $$s_{i}=\frac{\Delta x}{2}(\varepsilon_{0}+2\sum_{j}^{i-1}\varepsilon_{j}+\varepsilon_{i})\;$$

where *ε0* denotes the strain value for the first strain gauge near the loading end within the bonding area. Because two strain gauges were placed in parallel at the first position, the average value for those two strain gauges was assessed. The variable *ε_j_* (*j* = 1, 2···*i*) denotes the strain value for the *j*th strain gauge along the length direction of CFRP. The variables *E_f_* and *t_f_* denote the elasticity modulus and thickness of CFRP, respectively.

**Figure 11:**
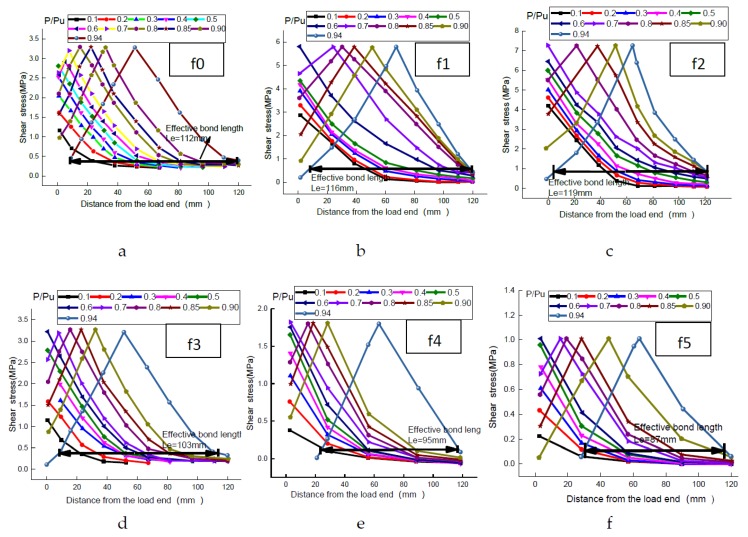
Shear strain–position diagram under different roughness. (a: f0; b; f1; c: f2; d: f3; e: f4; f: f5).

The effective bond length is an important parameter when studying the bonding performance of FRP-concrete interfaces. As the bonding length increased, the interface bearing capacity increased, correspondingly. Once it exceeded a certain fixed length $L_{e}$, even when bonding length continued to increase, interfacial bearing capacity remains unchanged. Such a fixed length $L_{e}$ is defined as an effective bond length [14]. Two methods can directly measure and calculate $L_{e}$ through test data: (1) as described by Le [15,16,17], measuring the distance between two points corresponding to 10% of the maximum bonding shear stress on the shear strain–position diagram; and (2) as described by S.A. Hadigheh et al. [18], measuring the distance between the points corresponding to 99% and 1% of the strain at the loaded end when the strain profile at the crack face tends to become plateau. In this paper, the first method was used, as shown in Figure 11. 

**Figure 12:**
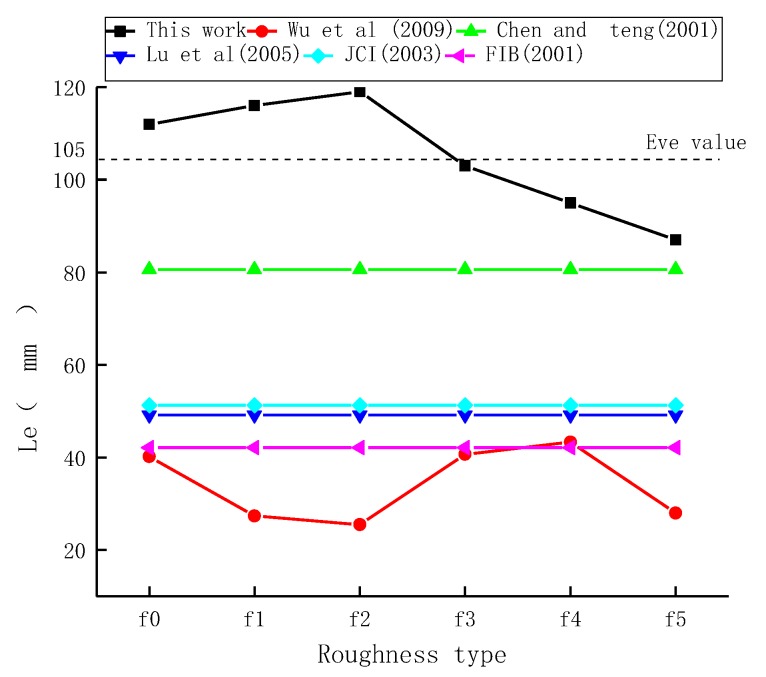
Comparison of effective bond length among different models.

As shown in Figure 11, in the range of f0–f5, effective bond length decreased overall as roughness increased. When interfacial roughness was considered, the effective bond length of interfaces all increased more significantly compared to existing models [19,20,21,22,23], as shown in Figure 12. Compared with the most commonly used model introduced by Lu et al. [19], the average effective bond length value of the six different surfaces with different roughness levels measured in tests was 113%, 127%, and 146% higher, respectively, under different concrete strength levels. This indicates that, when the roughness index for the FRP-concrete interface was considered, the rougher surface extended the storage space for interfacial energy and provided a larger bonding area. This improved interface bond strength. With the increase in the strength level of concrete (C30, C40 and C50), the effective bond length of interfaces increased. When the strength of the concrete increased by one level, the increased amplitude of the effective bond length was approximately 13%.

### 4.4. The Influence of Roughness on Interface Bond–Slip Curves

**Figure 13:**
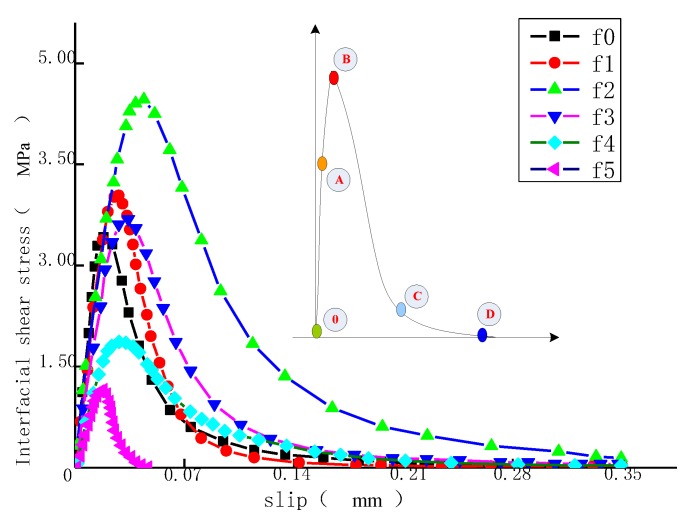
Interfacial constitutive relationship curve.

Figure 13 shows the constitutive relationship curves of the C40 concrete specimen with the interfaces with different roughness.

The bonding strength and the ultimate displacement of the interface are not always increased with the increase of interface roughness. However, this condition can be realized within certain limits. In Figure 13, the maximum interface bond stress is reached on f2 interface, with the bonding strength *τ*_m_ of 4.89 MPa and *s_τ_* of 0.035 mm. Six kinds of interface curves show the tendency increasing first and decreasing after; the whole curve can be divided into four intervals named: O–A, A–B, B–C, and C–D. In Interval O–A, the interface is in the situation of linear elastic tension. Reaching Point A, the epoxide resin comes into the plastic range, and stress increases slowly in Interval A–B, while slip grows. When Point B is reached, interface bond stress strengthens. Within the B–C range, epoxide resin continues to deform, and the deformation rate is bigger. At Point C, the interface shows the stress slowly increasing, and the epoxide resin deformation is still increasing. At Point D, the interfacial stress reaches zero, epoxide resin is thoroughly fractured, and the CFRP is debonded from the concrete surface. Three interfaces, f0–f3, are basically the same in the stiffness of elasticity area. Such property of these three interfaces can be overall deemed as the same. Interfaces f4–f5 are significantly decreased in stiffness, and the elasticity section is narrowed. Moving into the plastic stage, the bond stress–slip curves of six interfaces are overall declined in different slopes. Ultimately, these interfaces are torn in the slip value scope of 0.04–0.35 mm. It is acquired from the constitutive relation curve that the effective bond length is overall increased in the preliminary stage and decreased in the follow-up stage with the increase of roughness, which demonstrates the conclusion mentioned above.

## 5. Conclusions and Recommendations

An experimental investigation into the effects of concrete roughness on the bond shear strength of FRP-concrete interface was presented. Several concrete prisms, before strengthening, were prepared according to surface treatments; roughness level was then measured by means of sand filling method. Finally, specimens were subjected to single shear tests.

Fifty-four single shear tests were carried out between CFRP and concrete interfaces, and different stress–slip relations of interface under six types of roughness were acquired. Additionally, the influence of roughness carried by the concrete interface on the bonding shear property of FRP-concrete interface was analyzed. The following conclusions are drawn. 

Different surface preparations can provide different values of bond shear strength; in particular, the roughness value 0.44 measured by Chinese method is the most effective. An interfacial appearance with the cement mortar almost cleaned away, and almost one fifth of the single coarse aggregate bared will get the best bond performance.

Concrete surface damage can be greatly decreased when a layer of retarder is first gently coated on the bottom surface of the concrete specimen mold before the roughness processing. In fact, there are still many methods to measure contact, although the Chinese sand-filling method is an easy and efficient way to quantify the uneven surface.

It is not the case that the rougher the FRP-concrete interface, the greater its bonding strength and ultimate displacement. The laws governing variability only apply within a certain range.

The effective bond length of CFRP-concrete beam interface was significantly enhanced based on the roughness parameter. As roughness increased, the effective bond lengths of six interfaces all experienced an overall decreasing trend. 

Among the six interfaces, the interface with the roughness of 0.44 had the best bonding performance. The *τ*–*s* curves for interfaces with the roughness of 0.25–0.44 did not significantly differ in rigidity within the brittle region. However, the rougher an interface was, the shorter its brittle region was. After entering the brittle region, the bond–slip curves for six types of interfaces all declined with different slopes. The slip values were 0.04–0.35 mm when debonding failure occurred.

The present work confirmed again the important effects of roughness on the interface between CFRP and concrete. With only 162 test results, the sample is too small to derive a formula for engineers to use as a standard. Future research should enlarge the sample and propose a formula to calculate the bond strength, effective bond length, etc. The above values can be predicted based on the existing formula. Furthermore, the CFRP-concrete specimens should go through harsh environment such as high temperature, freeze–thaw cycles, seawater and alkaline solution, etc., as many elements in this research remain unchanged.

## Figures and Tables

**Table 1:** Concrete grade and mix proportion, kg/m^3^.

Strength Grade	Cement	Fly Ash	Coarse Aggregate (5–20 mm)	Fine Aggregate	Water Reducer	Water
C30	336	59	1045	789	3.95	167
C40	391	56	1051	580	3.47	165
C50	423	47	1104	660	6.11	150

**Table 2:** Physical parameters for material.

Material	Grade	Compressive Strength *f_c_*/MPa	Tensile Strength *f_t_*/MPa	Elastic Modulus *E_f_*/MPa	Thickness *t_f_*/mm	Area Weight *m_f_* /(g m^−2^)
Concrete	C30	35.0	5.3			
	C40	46.0	6.4			
	C50	57.5	7.4			
CFRP			3400.0	2.3 × 10^5^	0.167	300
Adhesive resin			38.0	2.4 × 10^3^		

**Table 3:** Quantized values for roughness of six types of interfaces.

Interfacial Specimen	f0	f1	f2	f3	f4	f5
Roughness	0.25	0.34	0.44	0.56	0.68	0.88

**Table 4:** Single shear test results.

Specimen	Roughness	Failure Mode/1,2,3	Fu/kN	$\tau_{m}/\mathrm{MPa}$	$s_{\tau }/\mathrm{mm}$	$s_{f}/\mathrm{mm}$
C30	f0	2	12.50	3.280	0.003	0.282
	f1	1,2,3	15.03	5.790	0.010	0.300
	f2	3	17.77	7.350	0.018	0.311
	f3	1	12.23	3.170	0.013	0.301
	f4	1	6.77	1.650	0.010	0.201
C40	f5	1	2.56	0.970	0.008	0.049
	f0	2	13.20	3.530	0.003	0.287
	f1	1,2,3	15.78	5.920	0.010	0.304
	f2	3	19.24	8.550	0.021	0.313
	f3	1	13.55	4.080	0.017	0.305
	f4	1	5.76	1.680	0.010	0.143
C50	f5	1	2.44	1.020	0.009	0.042
	f0	2	14.00	3.760	0.003	0.297
	f1	1,2,3	19.77	9.410	0.016	0.317
	f2	3	21.23	11.900	0.030	0.374
	f3	1	13.99	5.504	0.021	0.174
	f4	1	5.77	2.490	0.015	0.097
	f5	1	2.56	1.070	0.009	0.044

Note: Fu is the peak load of the interface between CFRP and concrete; $\tau_{m}$ is the interfacial strength; $s_{\tau }$ is the interfacial strength according to the $\tau_{m}$; and$s_{f}$ is the interfacial ultimate slip.

## Notation

fi	interface roughness
a	the length of the concrete’s bonding surface
b	and width of the concrete’s bonding surface
$E_{c}$	modulus of elasticity of concrete (MPa)
$E_{f}$	modulus of elasticity of CFRP sheet (MPa)
$m_{f}$	the mass unit area (g/m^2^)
*f_c_*	the compressive strength of the concrete (MPa)
*f_t_*	the tensile strength of the concrete (MPa)
*τ_m_*	interfacial bonding strength (MPa)
*s_τ_*	the slip value of corresponding *τ_m_* (mm)
*s_f_*	the maximum slip when interfacial bonding stress reduces to zero (mm)
*l_e_*	effective bonding length (mm)
Fu	maximum transferable load (bond capacity/bond strength) (kN)
*t_f_*	thickness of CFRP sheet (mm)
*s_i_*	local slip of the position *i* (mm)
*ε*_0_	the first strain value of the strain gauge next to the loading end
*ε_j_*	the strain value of the position *j* to the loading end

## References

[1] Ha S.K., Na S., Lee H.K. (2013). Bond Characteristics of Sprayed FRP Composites Bonded to Concrete Substrate Considering Various Concrete Surface Conditions. Compos. Struct..

[2] British Standard (2006). Common Rules for Precast Concrete Products.

[3] Chajes M.J., Finch W.W., Thomson T.A. (1996). Bond and force transfer of composite material plates bonded to concrete. Struct. J..

[4] Yao J., Teng J.G., Chen J.F. (2005). Experimental study on FRP-to-concrete bonded joints. Compos. Part B.

[5] Delaney J., Karbhari V. Defect criticality in FRP strengthening. Proceedings of the 8th International Symposium in Fiber-Reinforced (FRP) Polymer Reinforcement for Concrete Structures (FRPRCS-8) CD-ROM, University of Patras.

[6] Li W., Yan Z., Cao Z., Pan J. (2007). Effect of concrete surface roughness on the bonding performance between the CFRP and concrete. J. Shenzhen Univ. Technol..

[7] Zanpini D., Jennings H.M., Shah S.P. (1995). Characterization of the paste-aggregate interfacial transition zone surface roughness and its relationship to the fracture toughness of concrete. J. Mater. Sci..

[8] Garbacz A., Courard L., Kostana K. (2006). Characterization of concrete surface roughness and its relation to adhesion in repair systems. Mater. Charact..

[9] The Interior Bureau of Reclamation (1990). Concrete Handbook.

[10] JTG/T J22—2008. Highway bridges reinforcement design specification.

[11] Joost W., Agnieszka B.V. (2013). International Federation for Structural Concrete. Fib Model Code for Concrete Structures 2010.

[12] Zhao Z., Zhao G. (1999). Experimental research on treating interface of young-old concrete with high-pressure water jet method. J. Dalian Univ. Technol..

[13] Shang S., Yu D., Zhang R. (2010). Evaluation of surface roughness on strengthened RC structure. J. Build. Struct..

[14] Toutanji H., Han M., Ghorbel E. (2012). Interfacial bond strength characteristics of FRP and RC substrate. J. Compos. Constr..

[15] Nakaba K., Kanakubo T., Furuta T., Yoshizawa H. (2001). Bond behavior between fiber-reinforced polymer laminates and concrete. Struct. J..

[16] Fang E., Liu G., Zhang L. (2007). Experimental research on the bond performance of CFRP concrete interface. J. Build. Mater..

[17] Zhang P., Wu G., Zhu H., Meng S., Wu Z. (2014). Mechanical performance of the wet-bond interface between FRP plates and cast-in-place concrete. J. Compos. Constr..

[18] Hadigheh S.A., Gravina R.J., Setunge S. (2015). Prediction of the bond-slip law in externally laminated concrete substrates by an analytical based nonlinear approach. Mater. Des..

[19] Lu X. (2004). Studies on FRP-Concrete Interface. Ph.D. Thesis.

[20] Wu Z.S., Islam S.M., Said H. (2009). A three-parameter bond strength model for FRP—Concrete interface. J. Reinf. Plast. Compos..

[21] Chen J.F., Teng J.G. (2001). Anchorage strength models for FRP and steel plates bonded to concrete. J. Struct. Eng..

[22] Japan Concrete Institute (JCI) Technical report of technical committee on retrofit technology. Proceedings of the International Symposium on Latest Achievement of Technology and Research on Retrofitting Concretestructures.

[23] Thanasis T., Stijn M., Katrien A., György B., Michael B., Hendrik B., Christoph C., Emmanuelle D., Angello D.T., William D. (2001). Externally Bonded FRP Reinforcement for RC Structures.

